# Foot-and-mouth disease outbreaks in Egypt during 2013-2014: Molecular characterization of serotypes A, O, and SAT2

**DOI:** 10.14202/vetworld.2019.190-197

**Published:** 2019-02-05

**Authors:** Emad Diab, Abdel-Hamid I. Bazid, Mohamed Fawzy, Wagdy R. El-Ashmawy, Adel A. Fayed, Magdy M. El-Sayed

**Affiliations:** 1 Department of Internal Medicine and Infectious Diseases, Faculty of Veterinary Medicine, Cairo University, Cairo 12211, Egypt; 2 Department of Virology, Faculty of Veterinary Medicine, University of Sadat City, Menoufia 32958, Egypt; 3 Department of Virology, Faculty of Veterinary Medicine, Suez Canal University, Ismailia 41522, Egypt

**Keywords:** amino acids variations, Egypt, foot-and-mouth disease virus, FMDV, VP1 sequencing

## Abstract

**Background and Aim:**

Foot-and-mouth disease virus (FMDV) serotypes A, O and South African Territories (SAT2) are endemic in Egypt; each is presented by a number of partially related topotypes and lineages, depending on their geographical origin. Continuous mutations and the emergence of new topotypes that lead to occasional vaccination failures were frequently recorded, so this study aimed to genetically characterize the circulating FMD virus strains in Egypt during 2013 and 2014 outbreaks, focusing on amino acids variations in VP1 region.

**Materials and Methods:**

A total of 51 oral tissue samples were collected from cattle and buffaloes in 13 farms, and 38 individual cases showed clinical signs suspected to be FMD in six Egyptian Governorates (Cairo, Giza, Qaliubia, Fayoum, Sharquia, and Assiut). FMDV in collected samples was characterized by reverse transcription-polymerase chain reaction (RT-PCR) amplification of full VP1 region, sequencing, and phylogenetic analysis.

**Results:**

Out of 51 samples, 44 (86.27%) were positive by RT-PCR using universal primers. Serotype O was predominant and detected in 31 samples (70.45%), serotype A was detected in 9 samples (20.45%), and then serotype SAT2 was identified in 4 samples (9.10%). Sequencing and phylogenetic analysis of VP1 demonstrated clustering of serotype O, A, and SAT2 in EA-3 topotype, ASIA topotype, and topotype VII, respectively. Serotype O is closely related to O/SUD/8/2008 with 94.6% identity but showed 14.6% differences from vaccine strain (O/PanAsia-2) of ME-SA topotype. Furthermore, Serotype A and SAT2 were closely related to recent circulating Egyptian isolates and vaccine strains type A/EGY/1/2012 (Asia topotype, lineage Iran-05) with identity 96.4% and vaccine strain of SAT2/EGY/A/2012 (topotype VII, lineage SAT2/VII/ALX-12) with identity 95.3%, respectively.

**Conclusion:**

The present study recommended further studies of serotype O to determine the immunogenic relationship between the vaccine strain and the new strains to attain maximum protection against circulating viruses.

## Introduction

Foot-and-mouth disease is a highly contagious transboundary viral disease of cloven-hoofed animals with severe economic impact. The reason is the ability of the disease to cause loss of meat production, reduction of milk yield, abortions, prenatal mortalities, premature culling, restrictions of animal movement, vaccination cost, and treatment cost [1]. FMD is caused by a single strand positive-sense RNA virus which belongs to the genus Aphthovirus, family Picornaviridae [2]. The virus is small non-enveloped with icosahedral capsid symmetry containing 60 copies of four structural viral proteins (VP1-4), with VP1-3 exposed on the outside while VP4 is located internally [3]. The VP1 is the most variable protein among the capsid polypeptides and contains the major immunogenic epitopes responsible for neutralizing protective antibodies [4]. Phylogenetic analysis of VP1 has been used extensively to investigate the global foot-and-mouth disease virus (FMDV) molecular epidemiology [5]. FMDV has seven immunologically distinct serotypes: O, A, C, South African Territories [SAT1], SAT2, SAT3, and Asia1 with many antigenic variants in each serotype. Vaccination is an effective way to control FMD; however, the protection conferred by vaccination or infection is usually serotype specific and sometimes incomplete within a serotype [6].

Serotype O has a long history in Egypt with several outbreaks since 1951 [7-9]. Many topotypes and lineages were incriminated in these outbreaks including Sharquia-72 and PanAsia-2 lineages of Middle East-South Asia (ME-SA) topotype. In 2012, East Africa-3 topotype (EA-3) emerged and is still circulating [10]. Between 1964 and 2011, only serotype O was reported in Egypt, with the exception of years 1972 and 2006, when serotype A emerged due to importation of animals from African countries and resulted in the loss of one-third of Egyptian animal wealth [11]. On February 2006, 18 outbreaks were caused by serotype A, in which 6 outbreaks were in Ismailia Governorate (the site of virus entry), 2 outbreaks in Alexandria, 5 outbreaks in Dumyat, and one in each of Cairo, Behera, Dakahlia, Fayum, and Menoufia Governorates. By April 2006, additional 34 outbreaks were recorded, and phylogenetic analysis revealed the circulation of an African topotype [12]. However, in 2012, it was decided to employ the Asian topotype of serotype A (Iran-05 lineage) in the vaccination program rather than the Africa topotype [13,14]. Massive FMD outbreaks were reported in February 2012 due to the appearance of SAT2 serotype in Egypt [15]. Topotype VII was characterized in all locally recorded outbreaks, with some showing SAT2 topotype (I-XIV). The current inactivated trivalent FMD vaccine contains A, O, and SAT2 (topotype VII) serotypes [16]. These data have been documented by the World Reference Laboratory in Pirbright (http://www.wrlfmd.org/wrlfmd) [17].

The emergence of new serotypes or topotypes has been associated with the importation of animals from endemic African countries and use of incompletely matching vaccines, which made the animals prone to infections with antigenically atypical strains of FMDV [17,18]. Due to high mutation rate, a possibility of emerging of new FMDV strains, continuous monitoring, and genetic and antigenic characterization of circulating FMDV strains are crucial to ensure efficient vaccination and proper control measures [19]. Thus, this study aimed to characterize the circulating FMDV during outbreaks occurred in 2013-2014 and vaccine strains to assure the vaccine efficacy in Egypt.

## Materials and Methods

### Ethical approval

Samples collection from animals were reviewed and approved by the Animal Care and Use Committee (#141101E001) of the Middle East for Vaccines (ME VAC^®^), Sharquia, Egypt.

### Samples

A total of 51 oral tissue samples were collected from cattle and buffaloes in 13 farms and 38 individual cases that showed clinical signs suspected to be FMD in six Egyptian Governorates (Cairo, Giza, Qaliubia, Fayoum, Sharquia, and Assiut). The samples were collected during the FMD outbreaks from December 2013 to May 2014 and were placed in transport medium prepared according to the OIE [20] and delivered to Research and Development Department of ME VAC^®^, Sharquia, Egypt.

### Reverse transcription-polymerase chain reaction (RT-PCR)

Conventional RT-PCR was employed for detection and serotyping of FMD in 51 field samples, using QIAamp viral RNA mini kit (Cat# 52904, QIAGEN, Crawley, UK). The extracted RNA was first tested by universal FMDV primers, targeting 5 UTR region according to Reid *et al*. [21]. The RT-PCR was performed using Verso^™^ one-step RT-PCR kit according to the manufacturer instructions. Consequently, positive samples by universal RT-PCR were tested by serotype-specific primers for O and A according to Reid *et al*. [21] and SAT2 according to Ahmed *et al*. [14]. The PCR products were analyzed on 1.5% agarose gel containing 0.5 ug/ml of ethidium bromide (Sigma, USA). The DNA band was visualized by UV illumination, and the size was determined against DNA molecular weight marker (Fermentas, Germany).

### Sequencing and phylogenetic analysis

Eight PCR products were sent to MACROGEN for VP1 gene sequencing. The resulted sequences were trimmed and aligned with other representative sequences from GenBank and vaccine strains using BioEdit program version 7.2.5 software, IBIS Therapeutics, Carlsbad, CA and Clustal W 1.83 program (Bioinformatics, Swiss). These alignments were used to construct distance matrices using the Kimura 2-parameter nucleotide substitution model as implemented in the program MEGA version 6.0.1 [22]. Midpoint-rooted neighbor-joining trees were then constructed using program MEGA version 6.0.1. The robustness of the tree topology was assessed with 1000 bootstrap replicates as implemented in the program.

## Results

### RT-PCR

A total of 44 samples out of 51 were positive by RT-PCR using universal primers with 86.27%. Out of 44 positive samples, serotype O was the predominant serotype detected in 31 samples (70.45%), serotype A detected in 9 samples (20.45%), and then serotype SAT2 that found in 4 samples with 9.10% as shown in Table-1.

**Table-1:** FMDV detection and serotyping by RT-PCR.

Governorates	Universal positive/total tested	Serotype-specific RT-PCR		

		A	O	SAT2
Cairo	4/5	0	4	0
Giza	7/7	2	4	1
Qalubia	9/12	2	6	1
Fayoum	13/13	3	9	1
Sharquia	5/6	1	4	0
Assiut	6/8	1	4	1
Total	44/51	9	31	4

RT-PCR=Reverse transcription-polymerase chain reaction, FMDV=Foot-and-mouth disease virus

### VP1 gene sequence analysis

The complete VP1 coding region of 8 FMDV was amplified by RT-PCR using specific primers followed by sequencing. The results of VP1 gene sequencing for the analyzed PCR products indicate that there are different FMD serotypes circulating in Egypt as shown in Table-2.

**Table-2:** Locations, accession numbers, and names of sequenced FMDV.

Sample	Serotype	Governorate	Accession	Sequence name (serotype/L/EGY/YY)
1	A	Cairo	KR092701	Foot-and-mouth disease virus A/Cairo/EGY/2013
2	O	Qaliubia	KR261668	Foot-and-mouth disease virus O/Qaliubia/ EGY/2013
3	O	Giza	KR261669	Foot-and-mouth disease virus O/ Giza /EGY/2013
4	O	Fayoum	KR261670	Foot-and-mouth disease virus O/Fayoum/ EGY/2013
5	O	Giza	KR261671	Foot-and-mouth disease virus O/Giza /EGY/2013
6	O	Sharquia	KR261672	Foot-and-mouth disease virus O/Sharquia/ EGY/2014
7	O	Giza	KR261673	Foot-and-mouth disease virus O/Giza /EGY/2014
8	SAT2	Assiut	KR261674	Foot-and-mouth disease virus SAT2/Assiut/ EGY/2014

FMDV=Foot-and-mouth disease virus

### Phylogenetic analysis and amino acid variation of FMDV serotype A

**Figure-1:**
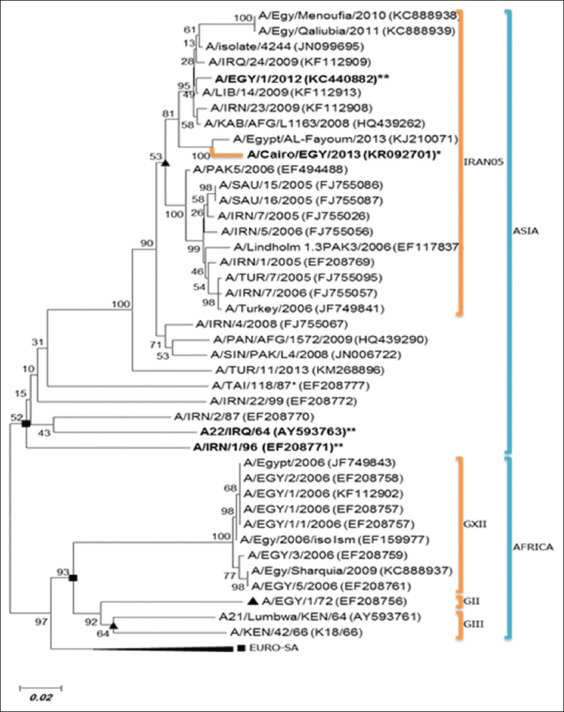
Phylogenetic tree of the VP1 sequence of foot-and-mouth disease virus (FMDV) serotype A isolated from Egypt. FMDV serotype A strain (bold&*) and Vaccine Strain (bold&**). The phylogenetic relationship bootstrap trial of 1000 was conducted using MEGA version 6, using clustal W alignment algorithm and neighbor-joining for tree construction.

A phylogenetic tree indicated that the detected FMDV (A/Cairo/EGY/2013 Accession #KR092701) belongs to A- Iran-05Bar-08 lineage of Asia topotype that differs from previously circulating Africa topotype as shown in Figure-1. The phylogenetic analysis clustered the characterized strain in the same clade of the vaccine strain, A/EGY/1/2012 (KC440882) with 96.4% nucleotide identity. Furthermore, this strain differs from the previously emerged African topotype strains of serotype A in Egypt which caused outbreaks in 1972, between 2006 and 2009 as A/EGY/1/72 (EF208756) and A/EGY/1/2006 (KF112902) with 79.1% and 78.2% of identity, respectively, as shown in Figure-1. The FMD/A/Cairo/EGY/2013 strain was closely related to recently circulating strains in the ME and Africa in 2009 and 2013 as A/Iran/4/2008 (FJ755067), A/LIB/14/2009 (KF112913), A/SAU/15/2005 (FJ755087), A/IRN/1/2005 (EF208769), and A/Egypt/Al-Fayoum/2013 (KJ210071) with percentage of identity 93.5%, 96.2%, 93.5%, 93.5%, and 98%, respectively.

**Figure-2:**
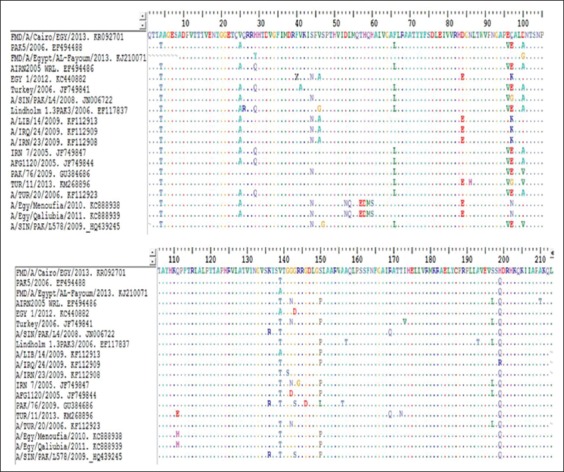
Amino acid alignment of serotype A showing differences (similarities shown as dots) in comparison with foot-and-mouth disease virus referaence strains present in GenBank and vaccine strains used in Egypt.

Figure-2 shows the alignment of the deduced amino acid sequences of the VP1 gene. Our isolate has the integrin binding motif “RGDLGSL” at 145-151 residues and alanine at residue 193, as well as a serine at residue 197. The most mutations were located at amino acid position 135-150 (at the G-H loop).

### Phylogenetic analysis and amino acids variation of FMDV serotype O

**Figure-3:**
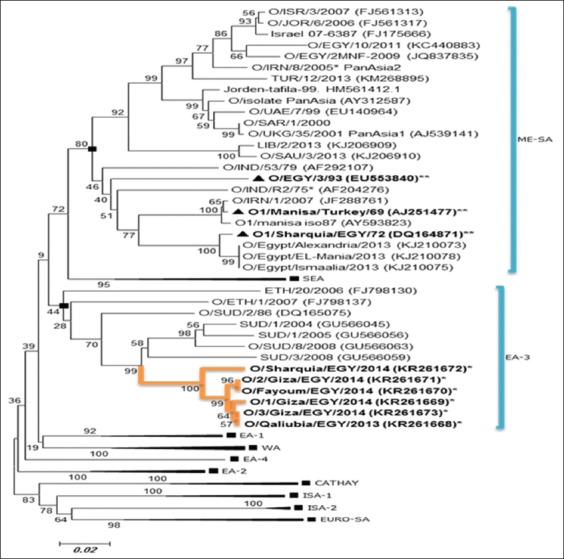
Phylogenetic tree of VP1 sequence foot-and-mouth disease virus (FMDV) serotype O isolates from Egypt and other countries. FMDV serotype O recent isolates (bold&*) and vaccine strain (bold&**). Phylogenetic relationship bootstrap trial of 1000 was conducted using MEGA version 6, using clustal W alignment algorithm and neighbor-joining for tree construction.

The sequencing results of 6 serotype O samples showed detection of a single strain belong to topotype East Africa 3 (EA-3). The characterized strains differ phylogenetically from Egyptian vaccine strains (O-PanAsia and O/EGY/93) of Sharquia-72 lineage within ME-SA topotype as shown in Figure-3. The results revealed that all 6 sequenced samples are closely related to each other as the identity ranged from 97% to 100% and also closely related to SUD/8/2008 (GU566063) by 94.6%. The results indicated that the all samples type O detected in this study were grouped in East Africa-3 (EA-3) topotype which detected in Egypt recently.

**Figure-4:**
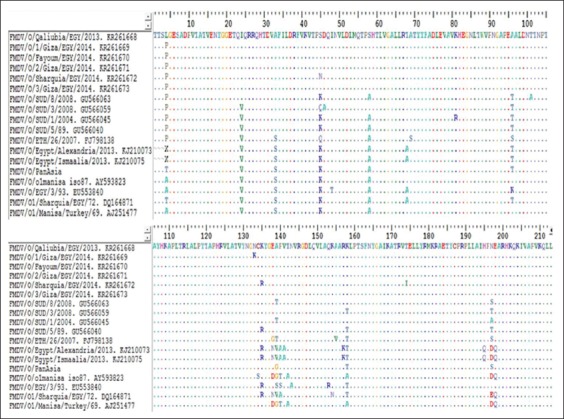
Amino acid alignment of serotype O showing differences (similarities shown as dots) in comparison with foot-and-mouth disease virus reference strains present in GenBank and vaccine strains used in Egypt.

To determine the distribution of mutations across the VP1 gene, amino acid variation was plotted, and regions of hypervariability were identified. These hypervariable regions were located at amino acid positions 131-149, 156-166, and 206-212 [Figure-4]. The former two hypervariable regions flank the RGD cell attachment site while the latter corresponds to another highly immunogenic portion of the VP1 gene, namely the C-terminal region. The cell attachment site Arg-Gly-Asp (RGD) at amino acid residues 145-147, within the G-H loop, was completely conserved across all O viruses included in this study. The leucine present at position 148 and cysteine residue at the base of the G-H loop (position 134) were conserved among all examined sequences [Figure-4].

### Phylogenetic analysis of FMDV serotype SAT2

**Figure-5:**
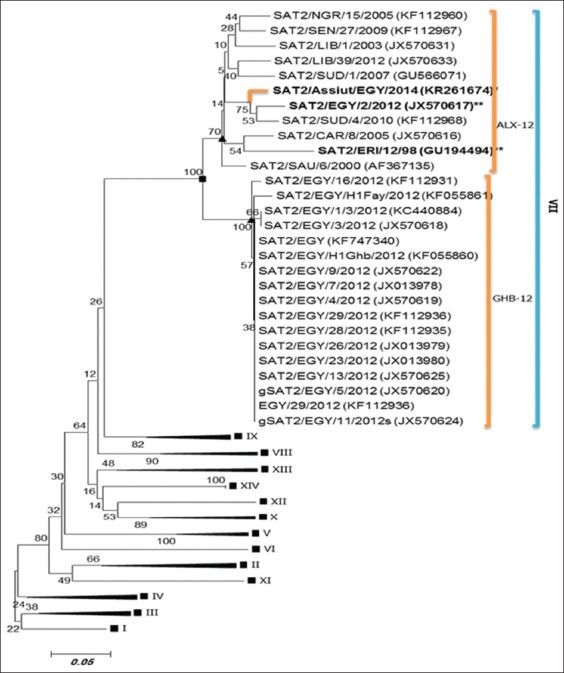
Phylogenetic tree of VP1 sequence foot-and-mouth disease virus (FMDV) serotype SAT2 isolates from Egypt and other countries. FMDV serotype SAT2 recent isolates (bold&*) and vaccine strain (bold&**). Phylogenetic relationship bootstrap trial of 1000 was conducted using MEGA version 6, using Clustal W alignment algorithm and neighbor-joining for tree construction.

The VP1 nucleotide sequence was determined for (SAT2/Assiut/EGY/2014 Accession #KR261674). This sequence was aligned and compared with sequences from GenBank including the Egyptian strains and vaccine strain used in Egypt. The results of the VP1 sequence analysis revealed that the sample was closely related to SAT2/EGY/2/2012 (JX570617) by 95.3% which is the vaccine strain. The characterized strains clustered in topotype VII, lineages ALX-12 as shown in Figure-5.

## Discussion

FMD molecular epidemiological studies can determine the genetic variation between FMDV circulating serotypes in Egypt. This genetic variation can partially explain the persistent occurrence of outbreaks even with obligatory governmental vaccination [12,23]. The VP1 genomic region that chosen for amplification contains the sequence for the major antigenic sites of the virus capsid (amino acid 138-160 of VP1) [24]. The present study described the genetic characterization of different FMDV serotypes which were the causative agent of FMD outbreaks in 6 Egyptian Governorates in 2013 and 2014.

The present study revealed circulation of FMDV serotype O which is the most predominant in Egypt followed by A then SAT2 serotypes. These results are in accordance with the World Reference Laboratory of FMD that described the predominant circulation of FMDV serotype O, topotype EA-3 since its first emergence in Egypt in 2012 [16].

Comparison of the obtained VP1 nucleotide sequence of serotype A (A/Cairo/EGY/2013 Accession #KR092701) with other isolates obtained from GenBank indicated that the detected isolate was closely related to vaccine strain (A/EGY/1/2012) of Asia topotype, Iran 05 Bar08. These results are in agreement with Sobhy *et al*. [25] who described serotype A (Asia topotype) but not agree with molecular characterization of Soltan *et al*. [10] who referred to it as serotype A (Africa topotype). The VP1 protein plays a major role in virus cell entry. The alignment of the deduced amino acid sequence of A/Cairo/EGY/2013 VP1 gene (isolated from clinical cases) with other isolates from GenBank showed that the VP1 contains amino acid alanine (A) at position 193. The alanine plays a role in heparan sulfate recognition, which is required for efficient infection of cell culture [26]. The presented results were agreed with Klein *et al*. [27] who reported that sequences derived from clinical cases or farms with a recent outbreak displayed an alanine instead of threonine at these positions. From the presented results of amino acid alignment of serotype A, we can conclude that the integrin binding motif of serotype A isolate A/Cairo/EGY/2013 was “RGDLGSL,” and this result agreed with that of Klein *et al*. [27] who reported that the serotype A Iran-05 isolated from clinical cases shows the integrin binding motif “RGDLGSL” while in subclinical cases, this motif was “RGDLGPL.” Unlike, any of the other common amino acids, proline has a cyclic ring, leading to a change in the secondary structure, and this change may play a role in the virus attachment efficiency.

Comparison of serotype O VP1 nucleotide sequence with those of other isolates in GenBank indicates that our isolates were closely related to O/SUD/8/2008; O/SUD/3/2008. They are fall in East Africa (EA-3) topotype that differs completely from the previous topotype ME-SA (lineage PanAsia-2) that was prevalent in Egypt from 2010 to 2012. These results were similar to Soltan *et al*. [10], Sobhy *et al*. [25], and Khodary *et al*. [28], who also detected serotype O, EA-3 topotypes. The amino acid variation was located at amino acid positions 131-149, 156-166, and 206-212. The first two hypervariable regions correspond the G-H loop while the latter corresponds the C terminal region of the VP1 [29]. The cell attachment site Arg-Gly-Asp (RGD) at amino acid residues 145-147, within the G-H loop, was completely conserved across all viruses included in this study. The presence of leucine at position 148 in serotype O enhances and stabilizes α-helix formation [30]. A cysteine residue at the base of the G-H loop (position 134) is associated with the disulfide-bond formation in serotype O viruses which is believed to promote conformational epitope formation [31].

FMD virus diversity is high among SAT serotypes, especially for the SAT2, and is composed of 14 topotypes [15]. FMDV serotype SAT2 outbreaks were officially reported in Egypt in 2012. Egyptian SAT2 virus is clustered into two distinct sublineages (ALX and GHB) within topotype VII [14]. In this study, the detected SAT2 sample belonged to SAT2 topotype VII within cluster ALX (SAT2/Africa/VII-Alex12). The detected isolate was closely related to SAT2/EGY/2/2012 of topotype VII; ALX-12 lineage and this confirmed by the phylogenetic tree. The obtained results agreed with Ahmed *et al*. [14], who mentioned that the outbreaks of SAT2 during 2012 were topotype VII that not detected previously in Egypt.

Finally, our phylogenetic analysis results are in agreement with Salam *et al*. [32] who found that type O belonged to ME-SA topotype (PanAsia-2 lineage), type A belonged to Asia topotype (Iran-05 lineage), and type SAT2 belonged to topotype VII.

## Conclusion

The genotyping data of Egyptian FMD viruses indicate multiple serotypes and genotypes circulating in Egypt; this heterogeneity is likely to be reflected antigenically. Hence, the present study recommends further studies for serotype O to confirm the immunogenic relationship between the vaccine strain and the new strains to provide maximum protection against circulating viruses.

## Authors’ Contributions

ED, AAF, and MME contributed equally to the design of the work. MF, ED, and AIB contributed equally to performing the work. ED and MF contributed to data analysis. MF, ED, and WRE contributed to sample collection. MF and ED contributed to writing the work. All authors read and approved the final manuscript.
